# Taxonomic and functional diversity of insect herbivore assemblages associated with the canopy-dominant trees of the Azorean native forest

**DOI:** 10.1371/journal.pone.0219493

**Published:** 2019-07-15

**Authors:** Carla Rego, Mário Boieiro, François Rigal, Sérvio P. Ribeiro, Pedro Cardoso, Paulo A. V. Borges

**Affiliations:** 1 cE3c - Centre for Ecology, Evolution and Environmental Changes/Azorean Biodiversity Group, Faculty of Agriculture and Environment, Department of Environmental Sciences and Engineering, Universidade dos Açores, Angra do Heroísmo, Açores, Portugal; 2 CNRS-Université de Pau et des Pays de l’Adour, Institut des Sciences Analytiques et de Physico-Chimie pour l'Environnement et les Materiaux, MIRA, Environment and Microbiology Team, UMR 5254, BP, Pau Cedex, France; 3 Laboratory of Evolutionay Ecology of Canopy Insects and Natural Succession/Instituto de Ciências Exatas e Biológicas, Universidade Federal de Ouro Preto, Ouro Preto, MG, Brazil; 4 Finnish Museum of Natural History, University of Helsinki, Helsinki, Finland; Universita degli Studi di Roma Tor Vergata, ITALY

## Abstract

Oceanic islands have been providing important insights on the structuring of ecological communities and, under the context of the present biodiversity crisis, they are paramount to assess the effects of biological invasions on community assembly. In this study we compare the taxonomic and functional diversity of insect herbivore assemblages associated with the dominant tree species of Azorean native forests and investigate the ecological processes that may have originated current patterns of plant-herbivore associations. Five dominant trees—*Erica azorica*, *Ilex perado* subsp. *azorica*, *Juniperus brevifolia*, *Laurus azorica* and *Vaccinium cylindraceum*—were sampled in the remnants of the native forest of Terceira Island (Azores) using a standardised methodology. The taxonomic and functional diversity of insect herbivore assemblages was assessed using complementary metrics and beta diversity partitioning analysis (species replacement and richness differences) aiming to evaluate the variation in insect herbivore assemblages within and between the study plant species. Sixty two insect species, mostly bugs (Hemiptera) and caterpillars (Lepidoptera), were found in the five study plants with indigenous (endemic and native non-endemic) insects occurring with higher species richness and abundance than introduced ones. Species replacement was the most important component of insect herbivore taxonomic beta diversity while differences in trait richness played a major role on functional beta diversity. The endemic *E*. *azorica* stands out from the other study plants by having associated a very distinct insect herbivore assemblage with a particular set of functional attributes, mainly composed by large bodied and long shaped species that feed by chewing. Despite the progressive biotic homogenization witnessed in the Azores during the last few decades, several strong associations between the endemic trees and their indigenous insect herbivores remain.

## Introduction

In recent time there has been an increasing effort to go beyond simple species richness when studying community structure by using complementary approaches such as indices of functional (FD) and phylogenetic diversity (PD) [1–3]. The FD of a given community is commonly quantified as the distribution in functional trait space of its species and individuals [4,5], and has thus emerged as a key concept in ecology to help deepen our understanding on community assembly processes and their association with ecosystem functioning. The growing use of FD relies on the fact that some morphological, physiological, behavioural or phenological traits affect, directly or indirectly, the performance and fitness of individuals [6]. Thus, contrary to taxonomic diversity (TD), the traditional way to analyse ecological diversity which relies on the assumption that all individuals and species are equal [7,8], FD accounts for the differences in traits between species and/or individuals and uses that information to characterize and interpret biodiversity patterns and processes. Furthermore, FD is a more straightforward way to study how biodiversity influences ecosystem functioning since directional relationships can be established between functional traits and the delivery of ecosystem processes [9,10].

Studies on functional diversity have been carried out in a wide variety of plant and animal groups, but mostly on vascular plants in which associations between particular traits and ecosystem processes have been set, helping to identify predictable trait-service clusters [10]. For example, most trait-based studies investigating plant-herbivore interactions explored how plant functional traits relate to herbivory pressure, but only a minority addressed the role played by phytophagous’ functional traits on herbivory levels and on insect-plant associations [11]. Thus, there is a need to extend the existing paradigms on plant functional diversity to other organisms, namely the insects that feed on them, as insect herbivores play a major role in regulating plant diversity and ecosystem functioning [12,13]. Patterns of local host plant use by herbivore communities have been studied in a variety of ecosystem-types worldwide [14–23], particularly in grasslands, and in temperate and tropical forests, but much more rarely on island ecosystems [24–26].

Oceanic islands are considered natural laboratories due to their discrete and replicated nature, thus providing the appropriate scenario to test ecological and evolutionary theories [27–28]. Nevertheless, to our knowledge, only a limited number of studies addressing the association of canopy insect taxonomic diversity with their host plants were carried out in oceanic island ecosystems [29–32]. When compared with continental settings, oceanic islands are usually poorer in animal and plant species and lack some important taxonomic and functional groups that are common in mainland [33,34]. These characteristics are particularly evident in the Azores, a relatively young and remote oceanic archipelago located in the North Atlantic [34,35]. The quite recent age of the Azorean islands and their remoteness, coupled with their history of volcanism along with modern human-driven disturbance, have been pointed out as major determinants for the low species richness and endemism in this archipelago [36–38]. Presently, the native biodiversity can mainly be found in the remaining forest patches on the different Azorean islands, where some microhabitats (like the canopies of endemic trees) accommodate a considerable fraction of native non-endemic and endemic species [39–41].

Previous studies on the insect fauna associated with the canopy of Azorean trees showed that the herbivore community is simple, being dominated by a few generalists and most species occur in low abundance [31,32]. The dominance of generalists in Azorean Laurisilva canopies may be due to relaxation of selection pressures since herbivore species richness and abundance is low, and then, competition for food is likely to be reduced. Thus, it has been argued that intra- and interspecific competition did not play a major role in structuring Azorean herbivore assemblages [25]. Further, the lack or low abundance of some canopy predators (e.g. ants, passerine birds) and parasitoids (e.g. chalcidoids, ichneumonoids) in Azorean native forests may have also favoured the prevalence of generalist herbivores since low selection pressure from higher trophic levels (i.e. top-down effect) allows larger host ranges in herbivores [35,42].

Nevertheless, evidence suggests that, besides species diversity and abundance, insect herbivore life-histories (e.g. specialist vs generalist) and chemically-mediated associational patterns may also play a role on the understanding of community assembly [43–46].

In this study, we compare the taxonomic and functional diversity of phytophagous insect communities associated with five dominant tree species of the Azorean native forests and evaluate the relative contribution of TD and FD ß-diversity components (species replacement and richness differences) to shed light on the ecological processes underlying community assembly. There have been only a few works which applied a functional trait-based approach to study biodiversity and ecological phenomena characteristic to island biota [34,47]. Moreover, we are unaware of any studies examining functional structure and trait-based assembly of phytophagous insects and their host plants on oceanic islands. Here we aim to contribute to fill this gap by specifically addressing the following questions: a) Do this five dominant tree species differ significantly in taxonomic and functional diversity of their associated insect herbivores?; b) what are the major processes (differences in species richness or species turnover) driving the changes in herbivore assemblages within and between plants?; and c) are there particular insect species and traits associated with each study plant? Based on previous work, we predict that plants with a more complex architecture (i.e. higher branching and leaf number) such as *E*. *azorica* and *J*. *brevifolia* should accommodate higher insect TD and FD than the other plant species. Furthermore, we hypothesize that there may be few associations of insect traits and species with each study plant since many Azorean herbivores have generalist diets.

## Materials and methods

Fieldwork for the collection of insect specimens was authorized by the Direção Regional dos Recursos Florestais dos Açores without the attribution of a permit number.

### Study area and plant species

The Azores are a volcanic archipelago composed by nine islands located in the middle of the North Atlantic Ocean and ranging between 37°-40°N and 25°-31°W. It is a relatively recent archipelago (0.3–8.1 Myr BP), quite far from mainland (over 1,500km from Europe and nearly 1,900km from North America), and presenting lower habitat diversity than other Macaronesian archipelagos [36,37,48,49]. The original vegetation of Azores included several types of native forest that dominated the landscape before human colonization, occupying all islands from the coast to the summit (up to 1,400m in Pico Island) [49,50]. The Azorean native forests are characterized by low tree stature (usually <5m) and high foliage density that contribute to low canopy openness and high levels of humidity. During the 600 years following human settlement in Azores, the native forests were severely destroyed and fragmented, and presently less than 5% of the original forest cover survives [50–52]. The Azorean native forest is currently absent from two islands (Corvo and Graciosa) and in the remaining seven it is restricted to high elevations (mostly above 600m).

The present study was conducted in Terceira Island, where the largest native forest fragments in the archipelago can still be found [51]. Nowadays only five fragments remain in this island: Biscoito da Ferraria, Guilherme Moniz, Pico Galhardo, Santa Bárbara and Terra Brava. Altogether, these five fragments occupy less than 6% of the island surface (covering 23.4km^2^), and due to their high value for nature conservation in the Azores were all included in a recently created natural park [53]. For our study, we chose the dominant tree species in Terceira native forests, the endemics *Erica azorica* Hochst. ex Seub., *Ilex perado* Aiton subsp. *azorica* (Loes.) Tutin, *Juniperus brevifolia* (Seub.) Antoine, *Laurus azorica* (Seub.) Franco and *Vaccinium cylindraceum* Sm. These five species show considerable functional and phylogenetic differences among them [31,54] (S1 Appendix). For instance, *E*. *azorica* and *J*. *brevifolia* have numerous small leaves and complex architectures while the other species have medium (*V*. *cylindraceum*) to large leaves (*L*. *azorica and I*. *perado*). Two of these species, *J*. *brevifolia* and *L*. *azorica*, are considered threatened in the IUCN red list due to their small area of occupancy and severely fragmented populations in the Azores [55,56]. The other study plants are not considered threatened and most of their populations lie within the limits of the Azorean network of protected areas.

### Insect sampling and functional trait collection

Insect sampling was undertaken in all the five fragments of Terceira´s native forest. Sampling effort (i.e. transect number) was proportional to fragment size with transects being randomly distributed in each fragment (S1 Table). The number of transects per forest fragment was established using a logarithmic scale, assuming a species-area relationship with a slope (z) of 0.35 in a log–log scale [31]. During the summers of 1999 and 2000, a total of 39 transects with 150m x 5m were set up to quantify species richness and abundance of herbivore insects on the dominant tree species of Terceira native forests. In each transect, ten individuals of the three most abundant tree species were sampled using a beating tray. The beating tray was placed beneath a randomly selected branch of a target plant species and the branch was hit five times with a stick to dislodge the arthropods (for further details see [31]). In some transects, where only one or two plant species were dominant, sampling was restricted to those that allowed the collection of ten replicates per plant species [31].

In this study, we focused on the insect groups that feed on plants either by sucking sap or by chewing leaves due to their ecological importance, easy sampling and well-known taxonomy in Azores. The other groups of phytophagous insects, such as leaf-miners and gall-inducers, were ignored as adequate sampling of these groups would require the application of additional sampling techniques and their identification is much more difficult due to the low knowledge on their biodiversity in Azores and the limited availability of taxonomic experts.

The sampling design allowed the collection of 1,250 individual samples from the five native forest fragments of Terceira. The samples are unevenly distributed by target plant species due to differences in species composition and relative abundance between transects and forest fragments (S1 Table). The samples were taken to the lab, where the insect specimens were sorted under the stereomicroscope and identified to species level whenever possible. All specimens are deposited in the “Dalberto Teixeira Pombo entomological collection” of the University of Azores (Angra do Heroísmo, Terceira, Azores).

The insect species were subsequently classified in distributional groups (endemic, native non-endemic, introduced or unknown). Morphospecies were classified considering the status of their close relatives in Azores or as unknown if information lacked (following the criteria adopted by Borges et al. [35]).

For each insect herbivore species, we gathered information on 23 trait attributes grouped in nine functional traits related to morphology, resource-use and dispersal. We collected information on body size, body shape, leg size and body toughness following the study of specimens under the stereomicroscope. The data on species locomotion, camouflage, period of activity, mode of ingestion and dispersal ability was recorded during fieldwork, collected from the literature [57–59] or obtained from experts. For morphospecies some traits were collected directly from specimens, but others were assigned considering the available information for close relatives (e.g. congeneric species). A detailed description of the nine functional traits along with their ecological relevance is given in Table 1.

**Table 1:** Functional traits used in the study and their ecological relevance. The data type of each trait variable is indicated jointly with the attributes. The ecological relevance of the selected traits is mentioned in several references ([47,51,59] and references therein).

Traits	Data type	Attributes	Ecological relevance
Body size	Continuous	Absolute body length in mm	Body size is related to life-history traits such as growth rate, clutch size and life span
Body shape	Multichoice nominal	Elongate, oval or long	Body shape is related to life-history traits associated with foraging and survival
Body toughness	Ordinal	Soft (1), medium (2) or hard (3)	Body toughness is related to life-history traits related with predation susceptibility
Leg size	Ordinal	Short (1), medium (2) or long (3)	Leg size is related with foraging activity
Locomotion	Ordinal	Walk (1), jump (2) or fly (3)	Locomotion is related with foraging activity
Camouflage	Binary	Yes (1) or no (0)	Camouflage is related with predation avoidance
Period of activity	Multichoice nominal	Day, night or twilight	Period of activity is related to life-history traits associated with the feeding ecology, predator avoidance and competitive interactions
Mode of ingestion	Nominal	Chewer or piercer	The feeding strategy is related to life-history traits associated with host plant use
Dispersal ability	Ordinal	Low (1), medium (2) or high (3)	Dispersal ability was based on wing development and influences foraging activity and colonisation potential

### Data analysis

#### Taxonomic and functional alpha diversity

For each plant species, we pooled the data on insects of the sampled individuals per transect and this was considered our unit of analysis. The first set of analyses aimed to test how phytophagous insects’ TD and FD differed between the five study plants. TD was assessed by the second-order Jackknife species richness estimator (Jack2) while species evenness was calculated using the Smith and Wilson’s index on log-transformed abundance data [60]. Insect herbivore species richness completeness of each study plant was assessed from the ratio of observed species richness to the values obtained from Jack2 richness estimator using the correction proposed by Lopez et al. [61]. Then, we assessed the differences on species richness completeness of the five study plants using a non-parametric Kruskal-Wallis one-way analysis of variance.

Preliminary to FD computation, we built the matrix of functional dissimilarity between species using the extension of the Gower’s distance which accommodates different types of variables [62]. Then, we measured the correlations between each pair of traits in order to estimate the level of redundancy in our functional information using Kendall rank correlations between every possible pair of distance matrices. Finally, to estimate the contributions of each trait to the global Gower’s distance, we applied the method proposed by Pavoine et al. [62].

Taking in consideration that FD comprises different facets of the variation in traits which together give a more complete picture of biodiversity [63], we calculated the following four FD metrics: functional richness, functional evenness, functional originality and functional specialization. Functional richness was assessed using a dendrogram-based approach whereby the unweighted pair group method with arithmetic mean (UPGMA) was used as the clustering algorithm [64] and was obtained from the sum of the length of all the branches required to connect all species in the community. True functional richness was subsequently estimated using the Jack2 estimator [65]. Functional evenness was assessed using the R_U_ index [66]. Functional originality and functional specialization (*sensu* [67]) were also computed, but since they are species-based measures, we derived a community-based measure of both metrics by calculating the abundance weighted average of the functional originality/specialization values of the species present in a given community.

To test whether TD and FD metrics differ between plants, we applied Linear Mixed Model analyses (LMMs) with plant species as a fixed effect and transects nested within forest fragments as a random effect to account for potential spatial effects and pseudo-replication. We quantified the effect of plant species using Magee's pseudo-R^2^ [68]. When the overall LMM was statistically significant, the Tukey’s post-hoc test was used to identify statistically significant pairwise differences between plants. To test the presence of functional assembly patterns across the five study plants, we compared the observed values of FD to a random distribution under the null hypothesis of no association between plants and traits. Therefore, we generated 999 null communities using the Independent Swap algorithm [69], which is known to minimize Type I error and has high power to detect trait-based processes in situations where multiple traits are involved [70]. To measure deviations from the null expectations for each value, we computed the standardized effect size (SES). We tested whether SES FD values for a given plant species presented significant deviations from the null expectation (median = 0) using one-sample Wilcoxon tests [71].

#### Taxonomic and functional beta-diversity decomposition analysis

To study the variation in the composition of phytophagous species assemblages within and between study plants, we performed a beta diversity partition analysis considering that:

$${T\beta }_{total}={T\beta }_{repl}+{T\beta }_{rich}$$

where Tβ_total_ represents the total variation in insect herbivore species composition between assemblages, Tβ_repl_ accounts for the variation due to species replacement, while Tβ_rich_ refers to the absolute differences in species richness [72,73].

Similarly, a beta diversity partitioning analysis was performed to assess variation in trait composition of insect herbivore assemblages within and between plant species. Using the functional UPGMA dendrogram, we partitioned total functional β (Fβ_total_) into its replacement (Fβ_repl_) and richness differences (Fβ_rich_) components following Cardoso et al. [74]. Both beta diversity decompositions (Tβ and Fβ) were computed using the Jaccard’s dissimilarity index and β measurements were weighted with log-transformed species abundances [65]. For Tβ and Fβ and their respective replacement and richness components, we first tested whether plant species had different variability in terms of species replacement and richness differences using a multivariate homogeneity of groups dispersions analysis (PERMDISP) [75]. Significance tests were performed with 999 permutations. When the overall PERMDISP was significant, Tukey HSD post-hoc tests were performed to identify statistically significant pairwise differences between plants. Subsequently, we conducted non-parametric permutational multivariate analyses of variance (PerMANOVA) to test for differences in species and trait composition between study plants [76] using permutation tests with 9999 iterations. When the overall PerMANOVA was significant, post-hoc t tests were performed to identify statistically significant pairwise differences between plants. Finally, Tβ and Fβ measures as well as their respective richness and replacement components were analyzed visually using constrained analysis of principal coordinates.

#### Association of insect herbivore species and their functional traits with host plants

We assessed the occurrence of indicator herbivore species for each study plant using the indicator value (IndVal) method [77]. This method is particularly useful since it combines information on ecological specificity and fidelity. In a similar way, we also tested which insect trait attributes were significantly associated with the study plant species using the point-biserial correlation coefficient (*r*_*pb*_) [78]. Point-biserial correlation coefficients were quantified on the community-weighted means (CWM) and computed separately for each trait. Multi-choice nominal traits and nominal traits were dummy-transformed to as many binary variables as there were trait attributes and were handled as quantitative variables [79]. For both IndVal and *r*_*pb*_, a permutation test (N = 9999) was used to determine whether particular species or traits were significantly associated with a given plant species under the null hypothesis of no relationship. All statistical analyses were implemented within the R programming environment [80] using the packages *indicspecies* [78], *vegan* [81], *nlme* [82] and *BAT* [65].

## Results

We sampled over ten thousand specimens from 62 species/morphospecies, particularly Hemiptera, Lepidoptera and Coleoptera (S2 Table). The first two insect orders were also the most represented in terms of species abundance, accounting for 97% of all collected specimens. The majority of the insect species sampled in Azorean trees is endemic or native non-endemic to the archipelago (67.7%) and introduced species are poorly represented in terms of species abundance (5.1%) (S3 Table).

### Sampling completeness and trait collinearity

Sampling completeness values are presented in S1 Fig. Average completeness values of insect herbivore species richness were in general high with small differences between plants (H = 10.85, P = 0.028, see S1 Fig). Therefore, we re-ran all our analyses (except species and functional richness estimation with Jack2) using rarefied assemblage matrices to test the sensitivity of our approach to the differences in completeness between sampling units. Since the results remained unchanged, we only present and discuss the analyses implemented on the original data. Trait collinearity was generally low. Only two traits, dispersal ability and period of activity, were highly correlated (S4 Table). The contribution of each trait to the global Gower functional distance matrix ranged from 0.33 for body toughness to 0.65 for the mode of ingestion.

### Patterns of taxonomic and functional alpha diversity

**Fig 1:**
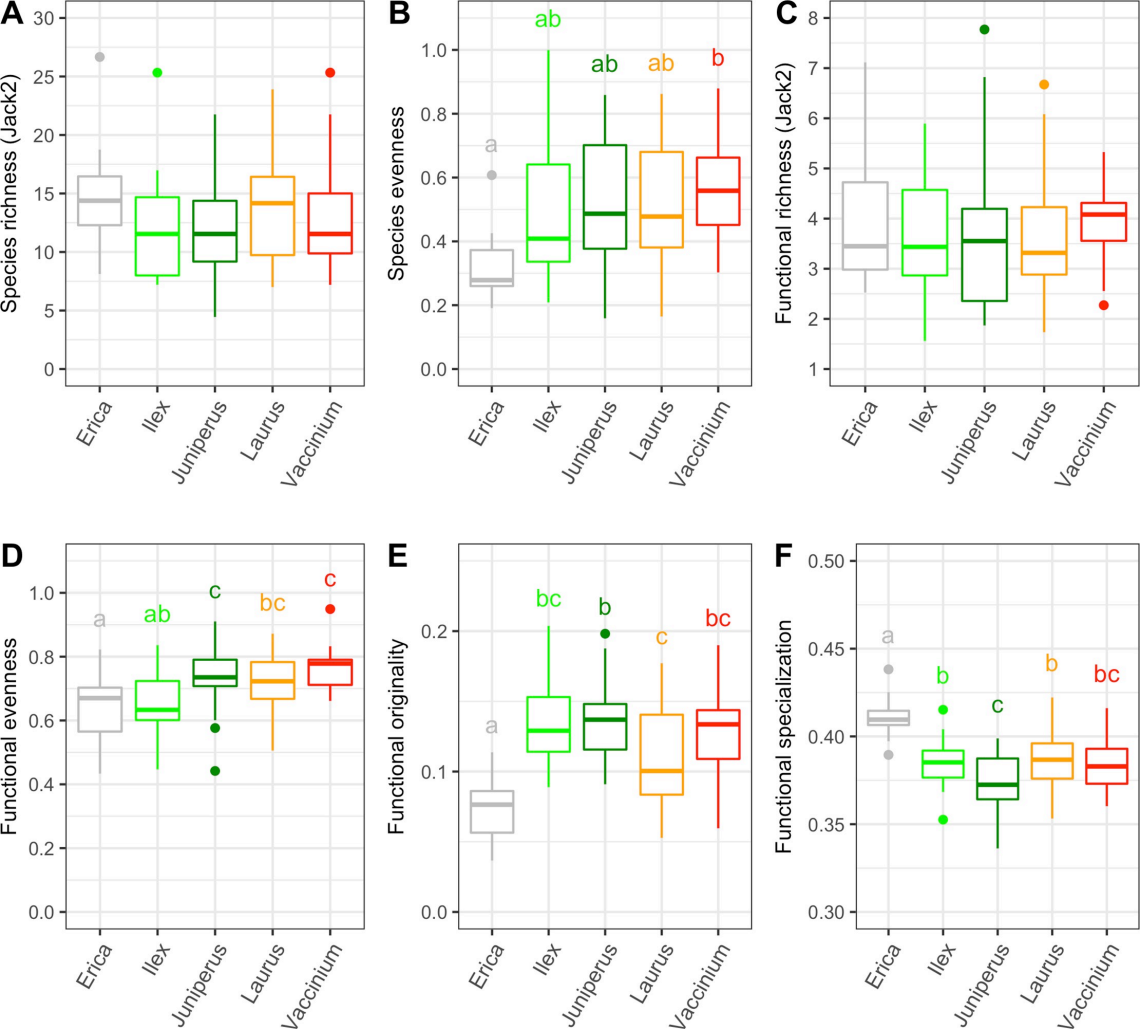
Taxonomic and functional diversity indices of insect herbivores associated with the different Azorean native forest trees. (A) Species richness estimated with Jackknife2, (B) species evenness measured with the index Evar, (C) functional richness estimated with Jackknife 2 (D) functional evenness measured with the index R_U_, (E) functional originality and (F) functional specialization. Significant differences of the diversity metrics between study plants were evaluated by linear mixed models followed by Tukey HSD tests. Study plants names were abbreviated to genus. Different letters associated with study plant names mean that they are significantly different from each other for that specific diversity metric.

Insect species and trait richness did not differ between plants, but significant differences were found in species and trait evenness and in functional originality and specialization (Fig 1, Table 2). *Erica azorica* stands out by presenting a very characteristic assemblage of herbivores with low balanced distributions of species and traits, low functional originality and high specialization (Fig 1). Minor but significant differences were also detected between the other study plants, particularly between *L*. *azorica* and *J*. *brevifolia*, with the former having lower functional originality and higher specialization than the latter. We found significant deviations from the null expectations for the four functional diversity metrics, suggesting the presence of non-random community assembly patterns across plant species (S5 Table). For instance, both *J*. *brevifolia* and *V*. *cylindraceum* displayed higher functional evenness than expected by chance (SES = 0.394 and 0.615, respectively; Wilcoxon test, P = 0.005 for both), *J*. *brevifolia* displayed higher functional originality (SES = 0.646, P<0.001) but lower functional specialization than expected by chance (SES = -0.582, P<0.001). In contrast, *E*. *azorica* showed lower functional originality (SES = -1.282, P<0.001) but higher functional specialization (SES = 0.973, P<0.001).

**Table 2:** Results of the linear mixed models analyses on the differences in the taxonomic and functional alpha diversity metrics of insect herbivores between the five study plant species.

	Diversity metrics	df	F	P	R^2^
Taxonomic diversity	Species richness (Jack2)	4,65	0.439	0.7797	0.04
	Species evenness	4,65	2.881	0.0293	0.10
Functional diversity	Functional richness (Jack2)	4,65	0.336	0.8525	0.13
	Functional evenness	4,65	6.184	<0.0001	0.19
	Functional originality	4,65	13.874	<0.0001	0.34
	Functional specialization	4,65	19.908	<0.0001	0.42

Degrees of freedom (df), F-statistics (F), associated p-values (P) and the Magee’s R^2^ are shown.

### Patterns of taxonomic and functional beta diversity

**Fig 2:**
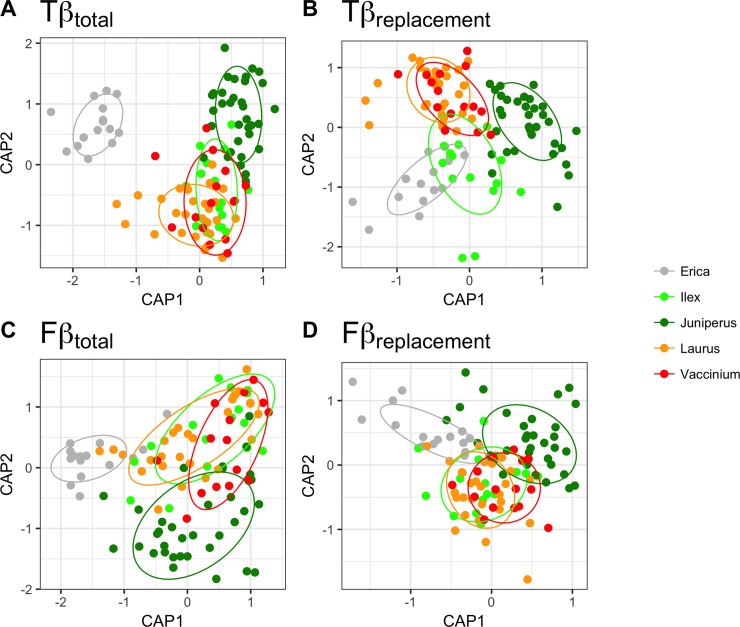
**Two-dimensional ordination based on the constrained analyses of principal coordinates for taxonomic (A, B) and functional (C, D) beta diversity.** (A) Tβ_total_ measured with Jaccard index; (B) Tβ_repl_, the component of Tβ_total_ that is due to species replacement between sites; (C) Fβ_total_ measured with Jaccard index incorporating branch lengths of the functional dendrogram and; (D) Fβ_repl_, the component of Fβ_total_ that is due to trait replacement between sites.

Taxonomic beta diversity of insect herbivores was high both between (Tβ_total_ = 0.75) and within (Tβ_total_ = 0.63) study plants (Fig 2, Table 3) and in both cases the major contribution was due to the species replacement component (Tβ_repl_, Table 3). The values of functional beta diversity of insect herbivores between and within study plants (Fβ_total_ = 0.56 and Fβ_total_ = 0.48 respectively) were lower than the corresponding taxonomic ones, with differences in trait richness being the most important component of beta diversity (Fβ_rich_, Table 3). For all beta diversity measures, PERMDISP analysis did not show significant differences in the amount of variability of herbivore species and trait composition between plant species except for Fβ_repl_ (S6 Table), where differences were observed between *E*. *azorica* and *I*. *perado* (Tukey HSD, P = 0.011). However, all PerMANOVA performed for each beta-metric were significant (all p<0.001, Table 4) indicating a clear overall difference in insect herbivore and trait composition between plants, with the highest R^2^ found for both Tβ_repl_ and Fβ_repl_. Specifically, for Tβ_repl_ and Fβ_repl_, post-hoc t tests revealed that all plant species were significantly different from each other suggesting a significant replacement of herbivore species and traits between plants (P<0.05, S7 Table). This pattern was particularly evident for *E*. *azorica* and *J*. *brevifolia*, with both presenting very characteristic compositions of both insect herbivore species (Tβ) and functional traits (Fβ) (Fig 2).

**Table 3:** Taxonomic and functional beta diversity of insect herbivores within and between study plant species.

	Beta metrics	Between plant species	Within plant species
Taxonomic diversity	Tβ_total_	0.75 ± 0.04	0.63 ± 0.04
	Tβ_repl_	0.44 ± 0.04	0.35 ± 0.04
	Tβ_rich_	0.31 ± 0.05	0.29 ± 0.03
Functional diversity	Fβ_total_	0.56 ± 0.05	0.48 ± 0.04
	Fβ_repl_	0.21 ± 0.01	0.17 ± 0.03
	Fβ_rich_	0.35 ± 0.05	0.31 ± 0.03

The results (mean ± SD) are presented for total taxonomic and functional beta diversity, showing also the contribution of the respective replacement and richness components.

**Table 4:** Results of the constrained analysis of principal coordinates (CAPSCALE) testing for differences in insect herbivore taxonomic and functional composition between study plant species.

	Beta diversity metrics	df	F	P	R^2^
Taxonomic diversity	Tβ_total_	4,65	8.519	0.001	0.221
	Tβ_repl_	4,65	9.169	0.001	0.285
	Tβ_rich_	4,65	2.490	0.022	0.044
Functional diversity	Fβ_total_	4,65	7.602	0.001	0.176
	Fβ_repl_	4,65	8.798	0.001	0.269
	Fβ_rich_	4,65	4.770	0.002	0.096

The results are presented for total taxonomic and functional beta diversity and their respective replacement and richness components. Degrees of freedom (df), F-statistics (F), associated p-values (P) and the R^2^ are shown.

### Association between insect herbivore species and functional traits with host plants

**Fig 3:**
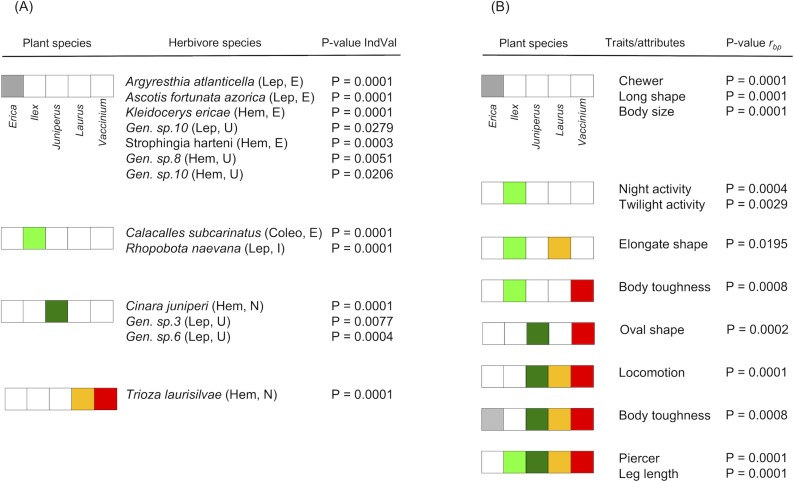
Insect herbivore and functional trait/attribute indicators for the five study plants. The order (Lep = Lepidoptera, Hem = Hemiptera and Coleo = Coleoptera) and the biogeographical origin (E = endemic, N = native non-endemic, I = introduced and U = Unknown) of each herbivore species/morphospecies is indicated. Functional traits and attributes were computed as CWM before analysis. Associations between plants and herbivore species were assessed using the IndVal index while the associations between plants and insect herbivore functional traits/attributes were evaluated with the point-biserial correlation coefficient *r*_*bp*_. Significant positive associations (using coloured squares) between host plants and insect herbivore species and traits are shown jointly with the significances of the results from permutation tests (P-value).

Thirteen phytophagous taxa were found to be significantly associated (i.e. indicator species) with the study plant species (Fig 3). Twelve indicator species were significantly and exclusively associated with single host plant species: seven insect herbivores were associated with *E*. *azorica*, three with *J*. *brevifolia* and two with *I*. *perado*. No insect herbivore species were found to be exclusively associated with *L*. *azorica* or *V*. *cylindraceum*, but these plants shared one indicator species, the sap-sucking psyllid *Trioza laurisilvae*. The indicator analysis for functional traits expressed as CWMs revealed that twelve traits or trait attributes were significantly associated with one or a combination of the five study plants (Fig 3). Chewers, large bodied species and species with elongated shape were significantly and exclusively associated with *E*. *azorica*, while night and twilight active species were exclusively associated with *I*. *perado*. Seven other traits or trait attributes were significantly associated with more than one plant species (Fig 3).

## Discussion

In this study, we investigated patterns of α and β taxonomic and functional diversity of insect herbivore assemblages from five dominant tree species of Azorean native forests. Although being widely recognized the key role played by insect herbivore species in the ecological and evolutionary dynamics of ecosystems [83], there are still few trait-based studies on this animal group on most ecosystems, particularly in oceanic islands.

### Patterns of taxonomic and functional alpha diversity

Our findings did not reveal significant differences in taxonomic and functional richness of insect herbivores between the five study plant species. However, the insect herbivore assemblage associated with *E*. *azorica* differed markedly from those of the other study plants by being taxonomically and functionally uneven. *Erica azorica* also differed from the other study plants by hosting an insect herbivore assemblage with lower functional originality and higher functional specialization. This finding unveils a degree of apparent functional redundancy in the insect herbivore assemblage of *E*. *azorica* with species being functionally similar to each other (i.e. low originality), but altogether occupying a very distinct portion of the functional space far from the centroid (i.e. high functional specialization). The results of the null model approach corroborated these findings by showing that functional originality was lower than expected by chance while functional specialization was higher than expected by chance. Consequently, it seems that the insect herbivore assemblage of *E*. *azorica* has been filtered out towards a specific functional profile, probably driven by the unique leaf morphology and architecture of the plant jointly with the influence of other factors (e.g. ecological and historical factors). The analysis of indicator traits (see section below) reinforced this finding by showing that the insect herbivore assemblage of *E*. *azorica* was specifically dominated by large bodied species with long shape and chewer feeding habits (e.g. caterpillars), differing markedly from the insect herbivore assemblages of the other study plants. Despite unrelated phylogenetically, the other study plants hosted insect herbivore assemblages that differed less between them in the taxonomic and functional diversity metrics. Three out of these four plant species show morphological and architectural affinities (see S1 Appendix) and they even shared a number of insect herbivores species, like the bugs *Cixius azoterceirae* and *Cyphopterum adcendens*.

The other plant species, *J*. *brevifolia*, has small leaves and a more complex architecture resembling *E*. *azorica*, but the associated insect herbivore assemblages of these two study plants differed significantly in functional originality and functional specialization. A possible explanation for those differences may be due to the much higher abundance of spiders found in *J*. *brevifolia* [32] which leads to higher predation risk on this plant, particularly for less mobile and soft-bodied insects. In fact, the prevalence of caterpillars on *E*. *azorica* as reported in this study may be a response to the enemy free space provided by this host plant, which has strongly influenced the results of functional specialization. However, we should not rule out the probability that the defense chemicals of *E*. *azorica* may have also played an important role as a driver of the insect herbivore assemblage. Plant chemistry can have profound bottom-up effects by influencing directly and/or indirectly the interactions with herbivores and their natural enemies as has been shown in many studies (e.g. [44–46,84–86]).Thus, further experimental studies considering the interspecific differences in plant chemistry are crucial for the understanding of the assembly of Azorean herbivore communities.

### Patterns of taxonomic and functional beta diversity

The taxonomic differences in insect herbivore assemblages between study plants were significant and mostly due to species replacement, not species richness differences (Fig 2, Table 4). Pairwise comparisons between plant species revealed a clear shift in the taxonomic composition with species replacement being highly significant between all plant species and even phylogenetically related plant species (i.e. the confamilial *E*. *azorica* and *V*. *cylindraceum*) showed very distinct herbivore assemblages (Fig 2, S7 Table). This finding is interesting since recent studies have warned for the progressive biotic homogenization witnessed in the Azores as a consequence of species introductions and native species loss due to forest destruction and fragmentation [87]. However, contrary to the findings on the epigean arthropod assemblages, the insect herbivore assemblages from the native forest canopies seem more resistant to human-driven impacts probably due to plant-mediated competitive interactions and/or habitat filtering effects [31,32,88,89]. A similar pattern was reported in a recent study [41] where the spider assemblages of native forest canopies were found to be largely dominated by indigenous species suggesting that these habitats may act as effective barriers for the colonization by exotic species.

In contrast to taxonomic β diversity, we found that trait replacement was twice lower than species replacement within and between plant species. Moreover, the contribution of trait replacement to the total functional β diversity was also much lower than the contribution of trait richness differences, irrespectively of plant species identity. This is a common pattern in many ecological studies as species performing similar functions replace each other between sites or time periods, reflecting a dynamic system where several functionally identical species may dominate at any point in space or time (e.g. [74,90]). Nevertheless, trait replacement, although low, was significant between all pairs of plant species highlighting the presence of a substantial functional turnover between the Azorean tree species (S7 Table). This finding suggests that trait filtering may operate between plant species driving trait-based assembly processes of insect herbivores in the canopies of Azorean native forests.

### Association between insect herbivore species and functional traits with host plants

The indicator analysis showed that one to a few insect herbivore species and traits are significantly associated with each study plant. *Erica azorica* was the plant species with a higher number of significantly and exclusively associated insect herbivores (seven species, 11% of the total sampled in *E*. *azorica*). Interestingly, four out of the seven species are endemics, including two caterpillars, *Argyresthia atlanticella* and *Cyclophora azorensis*, that are known to spend most of their development on *E*. *azorica* and to be responsible for substantial architectural damage in their crowns [88–91]. Trait indicator analysis for *E*. *azorica* identified trait attributes related with insect body form (long), size (big) and feeding mode (chewing), matching the findings of indicator species for this plant and being very distinct from insect attributes found in the other study plants.

Despite unrelated phylogenetically, *I*. *perado*, *L*. *azorica* and *V*. *cylindraceum* have more similar architecture and leaf form. In these plants, the characteristic insect herbivores were long-legged piercing bugs, but very few insect species were significantly associated with them. The native non-endemic bugs *Cinara juniperi* and *Trioza laurisilvae* are specialist species known to be associated with *Juniperus* species across Europe (the former) or to the Macaronesian *Laurus* species (the latter) and in Terceira native forests they exhibited the same host preference. Some insect herbivore traits associated with *I*. *perado* were shared with other study plants (e.g., long-legged, piercing) while others were exclusive to this plant (night and twilight activity) and matched the ecology of the two indicator species: the endemic weevil *Calacalles subcarinatus* and the introduced moth *Rhopobota naevana*. Night activity by insect herbivores is a functional trait commonly associated with predator avoidance since nocturnal foraging minimizes the probability of detection by many predators [92,93]. This behaviour is particularly important for insect herbivores feeding on plants with open canopy and large leaves (like *I*. *perado*) to avoid being exposed to predators during day-time and is frequently coupled with other predator avoidance mechanisms, like risk-sensitive foraging and diel microhabitat shifts.

Interestingly, the insect herbivore assemblage of *I*. *perado* showed a high proportion of introduced species (25.3%), contrasting markedly with the other study plants where introduced species accounted for less than 1% (except for *J*. *brevifolia* that reached 6.2%) (S3 Table). The introduced moth *Rhopobota naevana*, known to feed on holly (*Ilex aquifolium*) on its native range, has established in Azores being now strongly associated (i.e. indicator species) with the native congener *I*. *perado*. Host range expansion of phytophagous insects introduced in oceanic island ecosystems may become a serious conservation problem since the evolutionary change experienced by island plants (in traits related with resistance and defence) may render them to be highly susceptible to damage by alien herbivores. Nevertheless, considering our findings, there is no evidence of a present threat posed by exotic herbivores to the biodiversity and ecological processes on the Azorean native forest canopies.

Our findings showed that *E*. *azorica* (and to a less extent *J*. *brevifolia*) has a very distinct insect herbivore assemblage with several characteristic species and traits while other endemic trees, phylogenetically distinct but morphologically similar, shared several insect herbivore species and traits. In fact, βTD and βFD analyses revealed substantial levels of taxonomic and functional structure between study plants that, jointly with the indicator analyses, highlighted the distinctiveness of the insect herbivore assemblage of *E*. *azorica*. The other endemic trees (e.g. *I*. *perado*, *L*. *azorica* and *V*. *cylindraceum*) shared several native insect herbivore species which are known to have a wider trophic spectrum. Previous studies showed that generalist species are usually better represented on insect-plant interactions in island ecosystems when compared with the mainland ones [94,95]. For example, the prevalence of generalist pollinators on ecological networks has been reported from several Azorean islands [94,96,97] and similar findings were found for the insect herbivore assemblages associated with several plants across the archipelago [31,32]. These ecological patterns result from the poor dispersal capacity of some groups of insects, which seriously limits their probability of successfully colonizing oceanic islands. Consequently, in remote oceanic islands (like Terceira) the species poor assemblages of some insect groups experience the relaxation of interspecific competitive interactions and density compensation effects [94,95]. Furthermore, and specifically for insect herbivores, we also need to consider that low herbivory pressure during the early stages of plant species colonization and establishment on the islands may have favoured lower allocation of resources for secondary defences that might have benefited some herbivore species with wider trophic spectrum [98]. So, the low number of specialist herbivores in most of our study plants seems to reflect the remoteness and young geological age of Azorean islands and was likely aggravated during the last few decades by the severe habitat destruction that took place in the archipelago following human colonization. Specialist species are known to be more vulnerable to disturbance effects than generalists and are usually less able to survive in small habitat fragments subjected to frequent disturbance and encroachment by introduced species (e.g. [99]). In Azores, several large-bodied specialist species went extinct during the last century [100] and other endemic specialist species (including phytophagous beetles from genus *Drouetius*) are on the brink of extinction being considered conservation priorities [52,101,102].

Our analytical approach allowed us to tease out the relative contributions of the taxonomic and functional components involved in the trophic association between insect herbivores and their host plants, providing insights on the role of key species and traits that underlie an important ecosystem process. Additional research on the interactions between Azorean plants and their associated herbivores, particularly targeting the role of plant morphology, phylogeny and chemical ecology as drivers of species associations [44–46,103], is needed to further our understanding on the assembly of island communities.

## Supporting information

S1 Fig Differences in inventory completeness of the insect herbivores associated with the five study plants. (DOCX)

S1 Table Number of samples of each plant species collected in the different transects from the five native forest fragments in Terceira Island. (DOCX)

S2 Table Abundance of the insect species/morphospecies from different taxonomic groups on the five study plants. The reference number (Code) of voucher specimens deposited in the “Dalberto Teixeira Pombo entomological collection” of the University of Azores is also indicated.
(DOCX)

S3 Table Number of endemic, native non-endemic and introduced insect herbivore species and specimens found in the five study plant species from the Azorean native forests. (DOCX)

S4 Table Pairwise Pearson correlations between pairs of traits. (DOCX)

S5 Table Mean deviations from the null expectations for the four functional diversity metrics for each study plant. (DOCX)

S6 Table Results of the analysis of multivariate homogeneity of group dispersion (PERMDISP) performed on taxonomic and functional beta diversity of insect herbivores. (DOCX)

S7 Table Results of the post-hoc t tests of the significant PERMANOVAs testing for differences in taxonomic and functional beta diversity between plants. (DOCX)

S1 Appendix The study plants. Brief description and illustration of the study species.
(DOCX)
