# Supplementary material for: Taxonomic and functional diversity of insect herbivore assemblages associated with the canopy-dominant trees of the Azorean native forest
Source: PLoS One. 2019 Jul 15;14(7):e0219493. doi: 10.1371/journal.pone.0219493 (PMC6629062; doi:10.1371/journal.pone.0219493)
Supplement: S1 Fig — (DOCX) [file pone.0219493.s001.docx]

Inventory completeness

**S1 Figure. Differences in the inventory completeness of the insect herbivore species associated with the five study plants.**

The inventory completeness of each study plant was assessed by calculating the ratio of the observed insect herbivore species richness to the values of the second-order jackknife species richness estimator, using the correction of Lopez et al. [57]. Differences in completeness between study plants were tested using non-parametric Kruskal-Wallis one-way analysis of variance. The names of the study plants were abbreviated to their genus.
