# Supplementary material for: Taxonomic and functional diversity of insect herbivore assemblages associated with the canopy-dominant trees of the Azorean native forest
Source: PLoS One. 2019 Jul 15;14(7):e0219493. doi: 10.1371/journal.pone.0219493 (PMC6629062; doi:10.1371/journal.pone.0219493)
Supplement: S1 Table — (DOCX) [file pone.0219493.s002.docx]

**S1 Table. Brief description of the native forest fragments of Terceira Island and the number of samples from each plant species collected in the different transects. The description of the native forest fragments follows Gaspar et al. (2008) and Elias et al. (2016), where more detailed information can be found.**

| **Forest fragment name, location (UTM)**  **and description** | **Plant species** | **Number of**  **transects** | **Number of**  **samples** |
| --- | --- | --- | --- |
| Biscoito da Ferraria  UTM 26S 480849E 4290312N  This fragment is dominated by  “*Juniperus-Ilex* montane forests”and  “*Laurus* submontane forests”. | *Erica azorica* | 6 | 60 |
|  | *Ilex perado* subsp. *azorica* | 3 | 30 |
|  | *Juniperus brevifolia* | 8 | 80 |
|  | *Laurus azorica* | 5 | 50 |
|  | *Vaccinium cylindraceum* | 6 | 60 |
| Guilherme Moniz  UTM 26S 481480E 4284073N  This fragment is an early successional forest dominated by *E. azorica* and *L. azorica*. | *Erica azorica* | 4 | 40 |
|  | *Laurus azorica* | 4 | 40 |
|  | *Vaccinium cylindraceum* | 1 | 10 |
| Pico Galhardo  UTM 26S 480249E 4287288N  This fragment is dominated by  “*Laurus* submontane forests”. | *Ilex perado* subsp. *azorica* | 1 | 10 |
|  | *Juniperus brevifolia* | 4 | 40 |
|  | *Laurus azorica* | 4 | 40 |
|  | *Vaccinium cylindraceum* | 3 | 30 |
| Santa Bárbara  UTM 26S 472880E 4287368N  This fragment is dominated by “*Juniperus-*  *Ilex* montane forests”, “*Juniperus* montane woodlands” and “*Laurus* submontane forests”. | *Erica azorica* | 3 | 30 |
|  | *Ilex perado* subsp. *azorica* | 10 | 100 |
|  | *Juniperus brevifolia* | 16 | 160 |
|  | *Laurus azorica* | 9 | 90 |
|  | *Vaccinium cylindraceum* | 6 | 60 |
| Terra Brava  UTM 26S 482438E 4287412N  This fragment is dominated by  “*Juniperus-Ilex* montane forests”and  “*Laurus* submontane forests”. | *Erica azorica* | 2 | 20 |
|  | *Ilex perado* subsp. *azorica* | 3 | 30 |
|  | *Juniperus brevifolia* | 6 | 60 |
|  | *Laurus azorica* | 6 | 60 |
|  | *Vaccinium cylindraceum* | 2 | 20 |

Elias RB, Gil A, Silva L, Fernández-Palacios JM, Azevedo EB, Reis F. Natural zonal vegetation of the Azores Islands: characterization and potential distribution. Phytocoenologia. 2016; 46: 107–123.

Gaspar C, Borges PAV, Gaston KJ. Diversity and distribution of arthropods in native forests of the Azores archipelago. Arquipélago. Life and Marine Sciences. 2008; 25: 1–30.
