# Supplementary material for: Taxonomic and functional diversity of insect herbivore assemblages associated with the canopy-dominant trees of the Azorean native forest
Source: PLoS One. 2019 Jul 15;14(7):e0219493. doi: 10.1371/journal.pone.0219493 (PMC6629062; doi:10.1371/journal.pone.0219493)
Supplement: S2 Table — The reference number (Code) of voucher specimens deposited in the “Dalberto Teixeira Pombo entomological collection” of the University of Azores is also indicated. (DOCX) [file pone.0219493.s003.docx]

**S2 Table. Abundance of the insect species/morphospecies from different taxonomic groups on the five study plants. The reference number (Code) of voucher specimens deposited in the “Dalberto Teixeira Pombo entomological collection” of the University of Azores is also indicated.**

| **Order** | **Family** | **Species/morphospecies** | **Code** | ***Erica azorica*** | ***Ilex perado*** | ***Juniperus brevifolia*** | ***Laurus azorica*** | ***Vaccinium cylindraceum*** |
| --- | --- | --- | --- | --- | --- | --- | --- | --- |
| Coleoptera | Curculionidae | *Calacalles subcarinatus* (Israelson) | 141 | 0 | 51 | 8 | 15 | 5 |
| Coleoptera | Curculionidae | *Drouetius borgesi borgesi* Machado | 46 | 0 | 1 | 0 | 0 | 0 |
| Coleoptera | Curculionidae | *Phloeosinus gillerforsi* Bright | 568 | 0 | 1 | 0 | 0 | 0 |
| Coleoptera | Curculionidae | *Pseudophloeophagus tenax* (Wollaston) | 102 | 4 | 4 | 6 | 6 | 11 |
| Coleoptera | Curculionidae | *Sitona discoideus* Gyllenhal | 344 | 1 | 0 | 1 | 0 | 0 |
| Coleoptera | Nitidulidae | *Meligethes* sp | 896 | 1 | 0 | 0 | 0 | 0 |
| Coleoptera | Scraptiidae | *Anaspis proteus* (Wollaston) | 78 | 10 | 7 | 20 | 12 | 20 |
| Hemiptera | Aleyrodidae | Gen. sp.1 | 14 | 1 | 3 | 0 | 0 | 0 |
| Hemiptera | Aleyrodidae | Gen. sp.3 | 411 | 0 | 0 | 0 | 1 | 0 |
| Hemiptera | Aleyrodidae | Gen. sp.5 | 483 | 1 | 0 | 1 | 0 | 0 |
| Hemiptera | Aleyrodidae | Gen. sp.6 | 509 | 0 | 0 | 1 | 0 | 0 |
| Hemiptera | Aleyrodidae | Gen. sp.8 | 636 | 8 | 0 | 0 | 0 | 0 |
| Hemiptera | Aleyrodidae | Gen. sp.9 | 678 | 1 | 0 | 1 | 0 | 0 |
| Hemiptera | Aleyrodidae | Gen. sp.10 | 719 | 18 | 0 | 0 | 0 | 3 |
| Hemiptera | Aleyrodidae | Gen. sp.12 | 747 | 2 | 1 | 0 | 0 | 0 |
| Hemiptera | Aleyrodidae | Gen. sp.13 | 755 | 4 | 0 | 0 | 0 | 0 |
| Hemiptera | Aphididae | *Aphis craccivora* Koch | 106 | 0 | 0 | 1 | 0 | 0 |
| Hemiptera | Aphididae | *Covariella aegopodii* (Scopoli) | 362 | 0 | 0 | 0 | 0 | 1 |
| Hemiptera | Aphididae | *Dysaphis plantaginea* (Passerini) | 818 | 0 | 0 | 0 | 1 | 0 |
| Hemiptera | Aphididae | *Rhopalosiphum oxyacanthae* (Schrank) | 335 | 0 | 0 | 0 | 1 | 0 |
| Hemiptera | Aphididae | *Rhopalosiphum padi* (Linnaeus) | 171 | 0 | 1 | 0 | 0 | 0 |
| Hemiptera | Aphididae | *Rhopalosiphum rufiabdominalis* (Sasaki) | 320 | 0 | 0 | 0 | 1 | 1 |
| Hemiptera | Aphididae | *Uroleucon erigeronense* (Thomas) | 289 | 0 | 0 | 2 | 1 | 0 |
| Hemiptera | Cicadellidae | *Aphrodes hamiltoni* Quartau & Borges | 8 | 0 | 0 | 1 | 0 | 0 |
| Hemiptera | Cicadellidae | *Eupteryx azorica* Ribaut | 465 | 0 | 1 | 1 | 2 | 0 |
| Hemiptera | Cixiidae | *Cixius azoricus azoricus* Lindberg | 416 | 1 | 3 | 6 | 4 | 0 |
| Hemiptera | Cixiidae | *Cixius azoterceirae* Remane & Asche | 7 | 313 | 496 | 764 | 312 | 92 |
| Hemiptera | Coccidae | Gen. sp.1 | 58 | 0 | 0 | 1 | 0 | 0 |
| Hemiptera | Coccidae | Gen. sp.6 | 572 | 0 | 1 | 0 | 0 | 0 |
| Hemiptera | Coccidae | Gen. sp.7 | 714 | 0 | 0 | 0 | 2 | 0 |
| Hemiptera | Delphacidae | *Megamelodes quadrimaculatus* (Signoret) | 254 | 0 | 0 | 0 | 2 | 0 |
| Hemiptera | Flatidae | *Cyphopterum adcendens* (Herr.-Schaff.) | 124 | 67 | 181 | 324 | 232 | 218 |
| Hemiptera | Lachnidae | *Cinara juniperi* (De Geer) | 44 | 11 | 21 | 994 | 16 | 15 |
| Hemiptera | Lygaeidae | *Beosus maritimus* (Scopoli) | 782 | 1 | 0 | 0 | 0 | 0 |
| Hemiptera | Lygaeidae | *Heterogaster urticae* (Fabricius) | 904 | 0 | 0 | 1 | 0 | 0 |
| Hemiptera | Lygaeidae | *Kleidocerys ericae* (Horváth) | 167 | 678 | 3 | 4 | 128 | 6 |
| Hemiptera | Margarodidae | Gen. sp. | 487 | 1 | 0 | 5 | 0 | 0 |
| Hemiptera | Miridae | *Monalocoris filicis* (Linnaeus) | 476 | 1 | 2 | 1 | 6 | 1 |
| Hemiptera | Miridae | *Pinalitus oromii* J. Ribes | 137 | 72 | 26 | 178 | 53 | 65 |
| Hemiptera | Psyllidae | *Acizzia uncatoides* (Ferris & Klyver) | 662 | 2 | 0 | 0 | 1 | 0 |
| Hemiptera | Psyllidae | *Strophingia harteni* Hodkinson | 557 | 419 | 21 | 5 | 343 | 13 |
| Hemiptera | Triozidae | *Trioza laurisilvae* Hodkinson | 195 | 44 | 32 | 67 | 1293 | 123 |
| Lepidoptera | Geometridae | *Ascotis fortunata azorica* Pinker | 90 | 822 | 2 | 5 | 19 | 3 |
| Lepidoptera | Geometridae | *Cyclophora azorensis* (Prout) | 176 | 137 | 20 | 178 | 177 | 38 |
| Lepidoptera | Geometridae | *Nycterosea obstipata* (Fabricius) | 42 | 10 | 4 | 24 | 53 | 5 |
| Lepidoptera | Geometridae | *Xanthorhoe inaequata* (Warren) | 414 | 52 | 13 | 60 | 104 | 15 |
| Lepidoptera | Noctuidae | Gen. sp. | 424 | 0 | 1 | 0 | 0 | 0 |
| Lepidoptera | Tortricidae | Gen. sp.3 | 372 | 1 | 1 | 52 | 2 | 0 |
| Lepidoptera | Tortricidae | Gen. sp.6 | 410 | 3 | 1 | 108 | 2 | 1 |
| Lepidoptera | Tortricidae | Gen. sp.7 | 418 | 0 | 2 | 2 | 0 | 0 |
| Lepidoptera | Tortricidae | Gen. sp.10 | 519 | 11 | 0 | 0 | 1 | 0 |
| Lepidoptera | Tortricidae | *Rhopobota naevana* (Hübner) | 440 | 11 | 368 | 11 | 7 | 3 |
| Lepidoptera | Yponomeutidae | *Argyresthia atlanticella* Rebel | 19 | 465 | 0 | 52 | 21 | 31 |
| Lepidoptera |  | Gen. sp.3 | 375 | 9 | 0 | 0 | 3 | 2 |
| Lepidoptera |  | Gen. sp.9 | 603 | 0 | 1 | 1 | 1 | 0 |
| Lepidoptera |  | Gen. sp.18 | 754 | 0 | 0 | 1 | 3 | 0 |
| Lepidoptera |  | Gen. sp.22 | 906 | 0 | 0 | 0 | 0 | 1 |
| Lepidoptera |  | Gen. sp.23 | 907 | 0 | 0 | 0 | 0 | 1 |
| Thysanoptera | Phlaeothripidae | *Eurythrips tristis* Hood | 713 | 0 | 0 | 1 | 2 | 1 |
| Thysanoptera | Thripidae | *Aptinothrips rufus* Haliday | 359 | 0 | 0 | 0 | 1 | 0 |
| Thysanoptera | Thripidae | *Frankliniella* sp. | 903 | 0 | 1 | 0 | 0 | 0 |
| Thysanoptera | Thripidae | *Heliothrips haemorrhoidalis* (Bouché) | 276 | 1 | 0 | 0 | 0 | 1 |
