# Supplementary material for: Taxonomic and functional diversity of insect herbivore assemblages associated with the canopy-dominant trees of the Azorean native forest
Source: PLoS One. 2019 Jul 15;14(7):e0219493. doi: 10.1371/journal.pone.0219493 (PMC6629062; doi:10.1371/journal.pone.0219493)
Supplement: S3 Table — (DOCX) [file pone.0219493.s004.docx]

**S3 Table. Number of endemic, native non-endemic and introduced insect herbivore species and specimens found in the five study plant species from the Azorean native forests.**

|  | **Insect herbivore species richness** | | | |
| --- | --- | --- | --- | --- |
| **Plant species** | **Endemic** | **Native** | **Introduced** | **Total species** |
| *Erica azorica* | 13 | 13 | 8 | 34 |
| *Ilex perado* subsp. *azorica* | 12 | 12 | 6 | 30 |
| *Juniperus brevifolia* | 13 | 14 | 8 | 35 |
| *Laurus azorica* | 10 | 13 | 12 | 35 |
| *Vaccinium cylindraceum* | 9 | 12 | 5 | 26 |
| Total | 19 | 23 | 20 |  |
|  |  |  |  |  |
|  | **Insect herbivore abundance** | | | |
| **Plant species** | **Endemic** | **Native** | **Introduced** | **Total specimens** |
| *Erica azorica* | 2314 | 838 | 31 | 3183 |
| *Ilex perado* subsp. *azorica* | 649 | 300 | 321 | 1270 |
| *Juniperus brevifolia* | 1260 | 1450 | 178 | 2888 |
| *Laurus azorica* | 1050 | 1756 | 22 | 2828 |
| *Vaccinium cylindraceum* | 239 | 431 | 6 | 676 |
| Total | 5512 | 4775 | 558 |  |
