# Supplementary material for: Taxonomic and functional diversity of insect herbivore assemblages associated with the canopy-dominant trees of the Azorean native forest
Source: PLoS One. 2019 Jul 15;14(7):e0219493. doi: 10.1371/journal.pone.0219493 (PMC6629062; doi:10.1371/journal.pone.0219493)
Supplement: S4 Table — (DOCX) [file pone.0219493.s005.docx]

**S4 Table. Pairwise Pearson correlations between pairs of traits.**

|  | PA | BS | MI | BSH | CAM | LOC | DISP | LS |
| --- | --- | --- | --- | --- | --- | --- | --- | --- |
| BS | -0.026 |  |  |  |  |  |  |  |
| MI | 0.060 | 0.350 |  |  |  |  |  |  |
| BSH | -0.002 | 0.310 | 0.395 |  |  |  |  |  |
| CAM | 0.012 | 0.064 | 0.088 | 0.206 |  |  |  |  |
| LOC | 0.242 | 0.150 | 0.561 | 0.187 | 0.119 |  |  |  |
| DISP | 0.829 | -0.010 | 0.028 | -0.001 | 0.028 | 0.238 |  |  |
| LS | -0.044 | 0.228 | 0.366 | 0.425 | 0.075 | 0.226 | -0.072 |  |
| BT | 0.217 | -0.034 | 0.066 | 0.023 | 0.031 | -0.016 | 0.220 | -0.006 |

PA - Period of activity, BS - Body size, MI - Mode of ingestion, BSH – Body shape, CAM - Camouflage, LOC - Locomotion , DISP - Dispersal ability, LS – Leg size and BT - Body toughness.
