# Supplementary material for: Taxonomic and functional diversity of insect herbivore assemblages associated with the canopy-dominant trees of the Azorean native forest
Source: PLoS One. 2019 Jul 15;14(7):e0219493. doi: 10.1371/journal.pone.0219493 (PMC6629062; doi:10.1371/journal.pone.0219493)
Supplement: S5 Table — (DOCX) [file pone.0219493.s006.docx]

**S5 Table. Mean deviations from the null expectations of the four functional diversity metrics for each study plant.**

|  | Functional  richness | | Functional evenness | | Functional originality | | Functional specialization | |
| --- | --- | --- | --- | --- | --- | --- | --- | --- |
|  | mean SES | P | mean SES | P | mean SES | P | mean SES | P |
| *Erica* | -0.349 | 0.208 | -0.573 | 0.135 | **-1.282** | **<0.001** | **0.973** | **<0.001** |
| *Ilex* | **0.598** | **0.034** | -0.263 | 0.208 | 0.360 | 0.175 | 0.217 | 0.274 |
| *Juniperus* | -0.132 | 0.436 | **0.394** | **0.005** | **0.646** | **<0.001** | **-0.582** | **<0.001** |
| *Laurus* | -0.314 | 0.053 | 0.193 | 0.236 | -0.138 | 0.451 | -0.183 | 0.186 |
| *Vaccinium* | 0.422 | 0.151 | **0.615** | **0.005** | 0.245 | 0.229 | -0.162 | 0.359 |

Deviations from the null expectations were computed using the standardized effect size (SES). For each study plant species, the significance of deviations from the null expectations (median=0) were tested using one-sample Wilcoxon tests. The mean SES and the P-value (P) of the Wilcoxon test are given for each study plant and the significant results are marked in bold. The names of the study plants were abbreviated to their genus.
