# Supplementary material for: Taxonomic and functional diversity of insect herbivore assemblages associated with the canopy-dominant trees of the Azorean native forest
Source: PLoS One. 2019 Jul 15;14(7):e0219493. doi: 10.1371/journal.pone.0219493 (PMC6629062; doi:10.1371/journal.pone.0219493)
Supplement: S6 Table — (DOCX) [file pone.0219493.s007.docx]

**S6 Table. Results of the analysis of multivariate homogeneity of group dispersion (PERMDISP) performed on the taxonomic and functional beta diversity of insect herbivores.**

|  | Beta metrics | df | F | P |
| --- | --- | --- | --- | --- |
| Taxonomic diversity | Tβ_total_ | 4,103 | 1.639 | 0.17 |
|  | Tβ_repl_ | 4,103 | 0.878 | 0.48 |
|  | Tβ_rich_ | 4,103 | 0.424 | 0.791 |
| Functional diversity | Fβ_total_ | 4,103 | 1.244 | 0.297 |
|  | Fβ_repl_ | 4,103 | 3.439 | **0.011** |
|  | Fβ_rich_ | 4,103 | 0.388 | 0.817 |

The results are presented for total taxonomic and functional beta diversity as well as the contribution of the respective replacement and richness components. The degrees of freedom (df), the F-ratio (F) and the associated P-values (P) are given. Significant results are marked in bold.
