# Supplementary material for: Taxonomic and functional diversity of insect herbivore assemblages associated with the canopy-dominant trees of the Azorean native forest
Source: PLoS One. 2019 Jul 15;14(7):e0219493. doi: 10.1371/journal.pone.0219493 (PMC6629062; doi:10.1371/journal.pone.0219493)
Supplement: S7 Table — (DOCX) [file pone.0219493.s008.docx]

**S7 Table. Results of the post hoc t tests of the significant PERMANOVAs testing for differences in the taxonomic and functional composition of insect herbivores between the study plant species.**

| **Tβ_total_** |  | *Erica* | *Ilex* | *Juniperus* | *Laurus* |
| --- | --- | --- | --- | --- | --- |
|  | *Ilex* | **0.001** |  |  |  |
|  | *Juniperus* | **0.001** | **0.001** |  |  |
|  | *Laurus* | **0.001** | **0.001** | **0.001** |  |
|  | *Vaccinium* | **0.001** | **0.001** | **0.001** | **0.002** |
| **Tβ_repl_** |  | *Erica* | *Ilex* | *Juniperus* | *Laurus* |
|  | *Ilex* | **0.001** |  |  |  |
|  | *Juniperus* | **0.001** | **0.001** |  |  |
|  | *Laurus* | **0.001** | **0.001** | **0.001** |  |
|  | *Vaccinium* | **0.013** | **0.001** | **0.001** | **0.013** |
| **Tβ_rich_** |  | *Erica* | *Ilex* | *Juniperus* | *Laurus* |
|  | *Ilex* | **0.021** |  |  |  |
|  | *Juniperus* | **0.034** | 1 |  |  |
|  | *Laurus* | **0.034** | 1 | 1 |  |
|  | *Vaccinium* | **0.002** | 1 | 1 | 1 |
| **Fβ_total_** |  | *Erica* | *Ilex* | *Juniperus* | *Laurus* |
|  | *Ilex* | **0.001** |  |  |  |
|  | *Juniperus* | **0.001** | **0.002** |  |  |
|  | *Laurus* | **0.001** | 0.059 | **0.001** |  |
|  | *Vaccinium* | **0.001** | 0.059 | **0.002** | 0.059 |
| **Fβ_repl_** |  | *Erica* | *Ilex* | *Juniperus* | *Laurus* |
|  | *Ilex* | **0.011** |  |  |  |
|  | *Juniperus* | **0.001** | **0.001** |  |  |
|  | *Laurus* | **0.001** | **0.008** | **0.001** |  |
|  | *Vaccinium* | **0.006** | **0.009** | **0.003** | **0.033** |
| **Fβ_rich_** |  | *Erica* | *Ilex* | *Juniperus* | *Laurus* |
|  | *Ilex* | **0.008** |  |  |  |
|  | *Juniperus* | **0.008** | 1 |  |  |
|  | *Laurus* | **0.013** | 1 | 1.000 |  |
|  | *Vaccinium* | **0.002** | 1 | 0.623 | 1 |

PERMANOVAs were performed to test for differences in taxonomic and functional beta diversity metrics (Tβ and Fβ) and their species richness and replacement components between the five study plants. Significant results are marked in bold. The names of the study plants were abbreviated to their genus.
