# Supplementary material for: Taxonomic and functional diversity of insect herbivore assemblages associated with the canopy-dominant trees of the Azorean native forest
Source: PLoS One. 2019 Jul 15;14(7):e0219493. doi: 10.1371/journal.pone.0219493 (PMC6629062; doi:10.1371/journal.pone.0219493)

**Supporting Information**

Carla Rego, Mário Boieiro, François Rigal, Sérvio P. Ribeiro, Pedro Cardoso, Paulo A.V. Borges^.^ Taxonomic and functional diversity of insect herbivore assemblages associated to the canopy-dominant trees of the Azorean native forest.

**S1 Appendix. The study plants.** Brief description and illustration of the study species.

*Erica azorica* – small evergreen tree (up to 6m) with a complex architecture and simple needle-like leaves. Occurs in all Azorean islands, particularly in coastal cliffs (*Erica*-*Morella* coastal woodlands), lava flows and dry slopes, where behaves as an early successional plant. Can also be found associated with mid elevation native forests, occurring up to 1500 m.


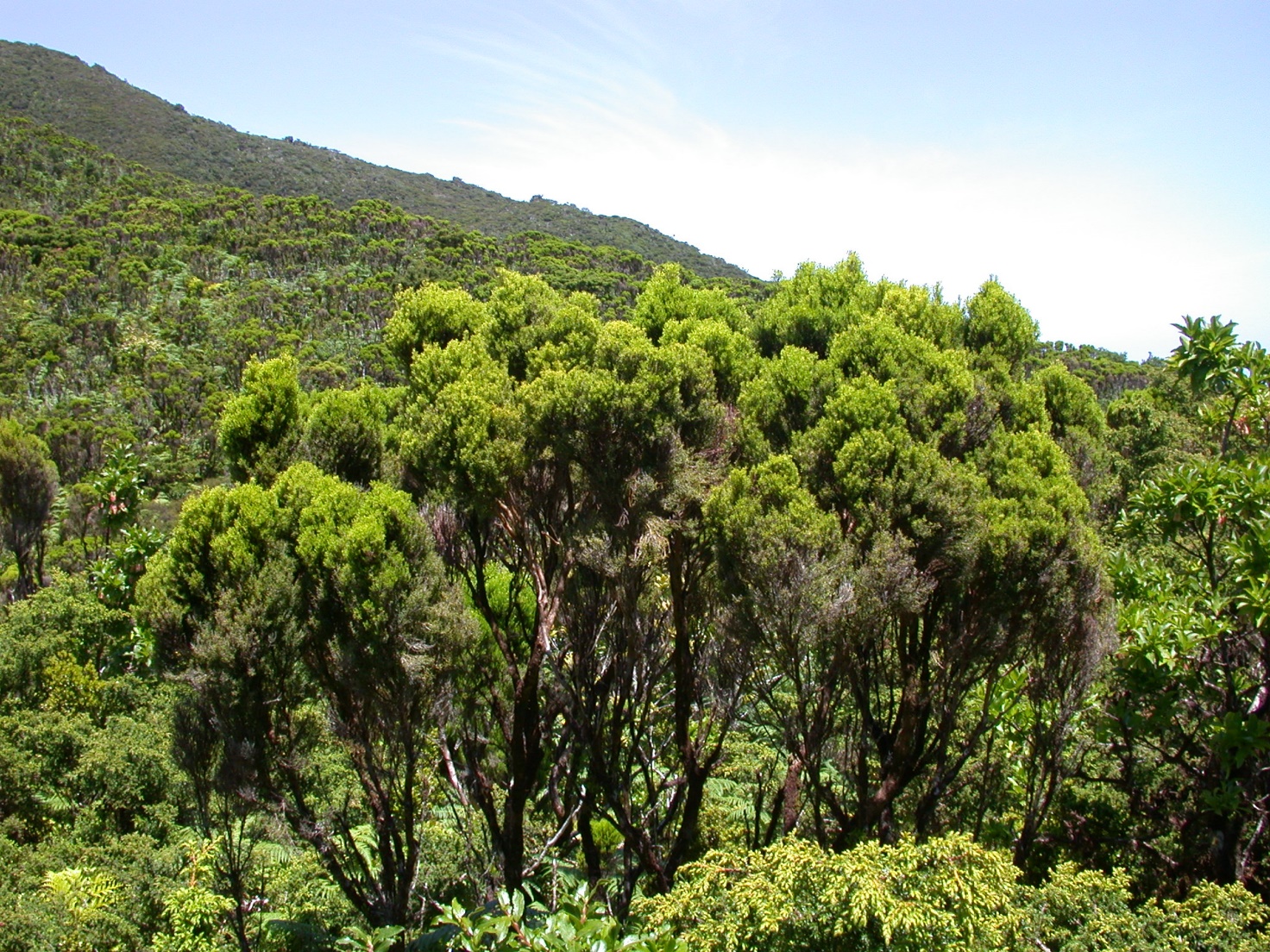


*Ilex perado* subsp. *azorica* – small evergreen tree (up to 7m) with a smooth bark. Leaves are simple, glabrous and spiny, with up to 6 cm. Occurs in ravines and forest patches (*Juniperus*-*Ilex* forests) in eight Azorean islands (excluding Graciosa) between 250 and 750 m.


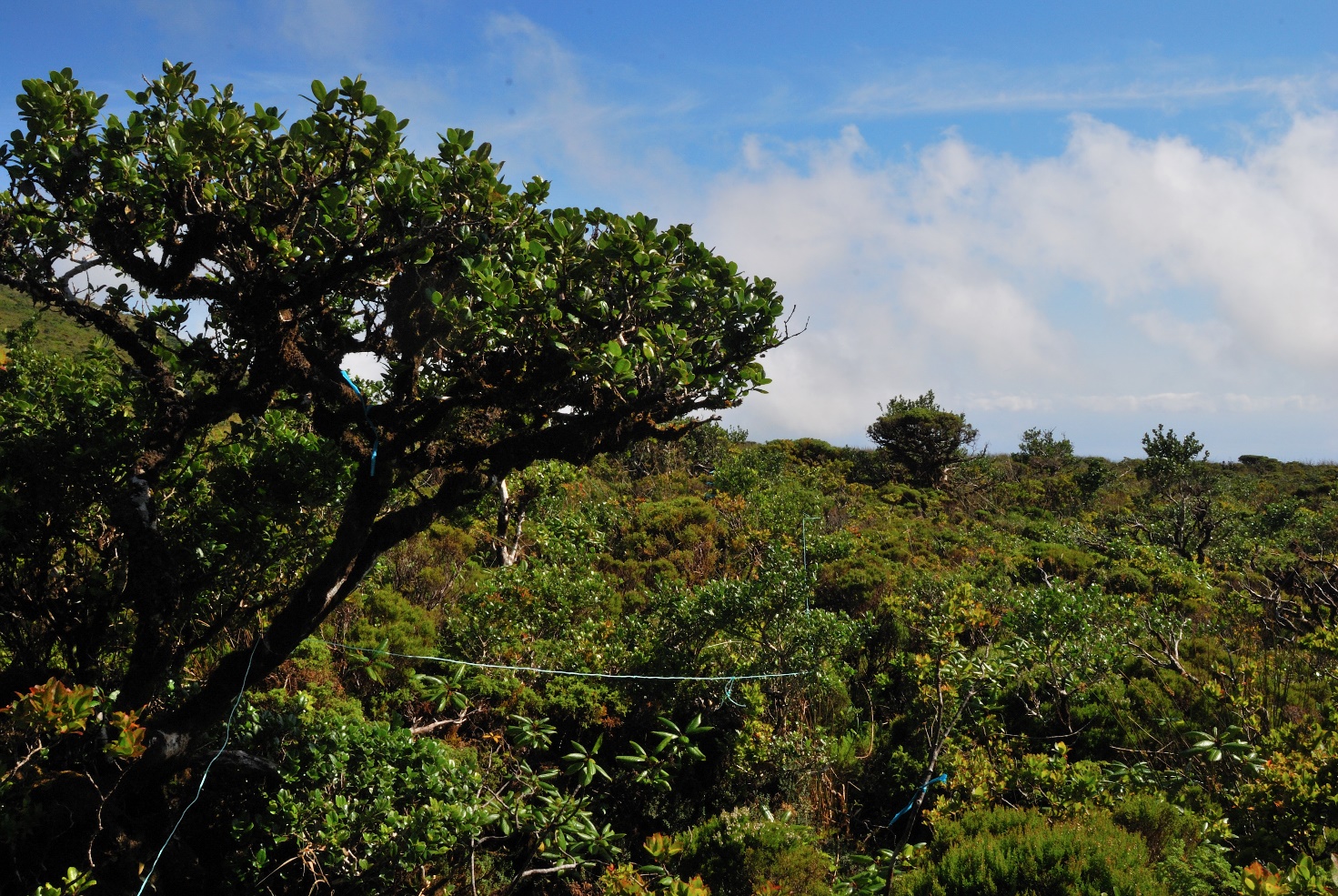


*Juniperus brevifolia* – evergreen tree (up to 12m) with a complex architecture and needle-like leaves. Trunk bark brown-purple, exfoliating in strips. Occurs in eight Azorean islands (extinct in S. Maria), mostly at medium to high elevations, being currently the dominant tree in some forests and woodlands between 500 and 1000 m.


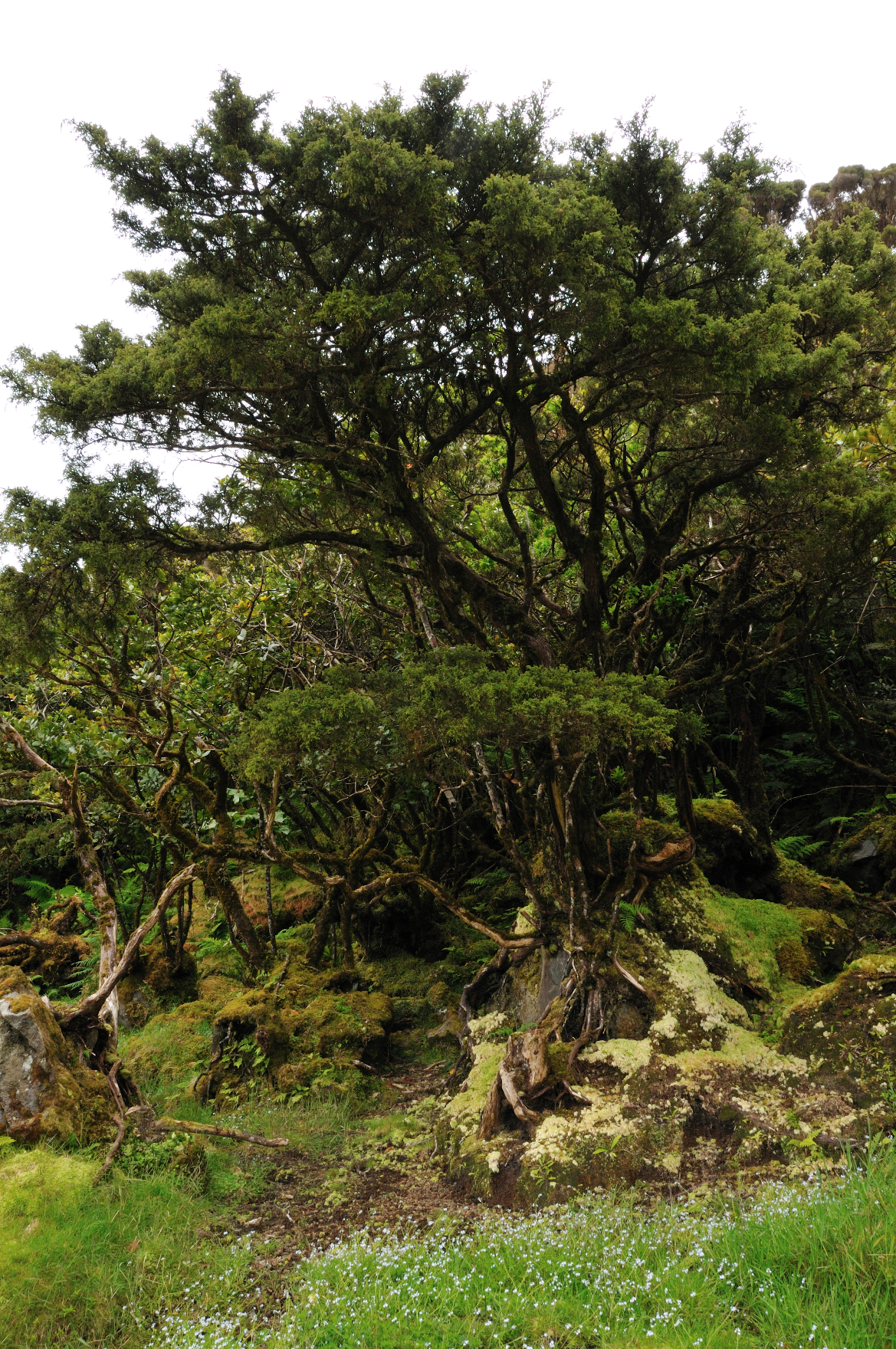


*Laurus azori*ca – evergreen tree (up to 13m) with large, elliptic, oblong or ovate, glabrous leaves (up to 15cm) and with a smooth bark. Occurs in all Azorean islands and can be found on steep slopes and forest patches (*Laurus* submontane forests).


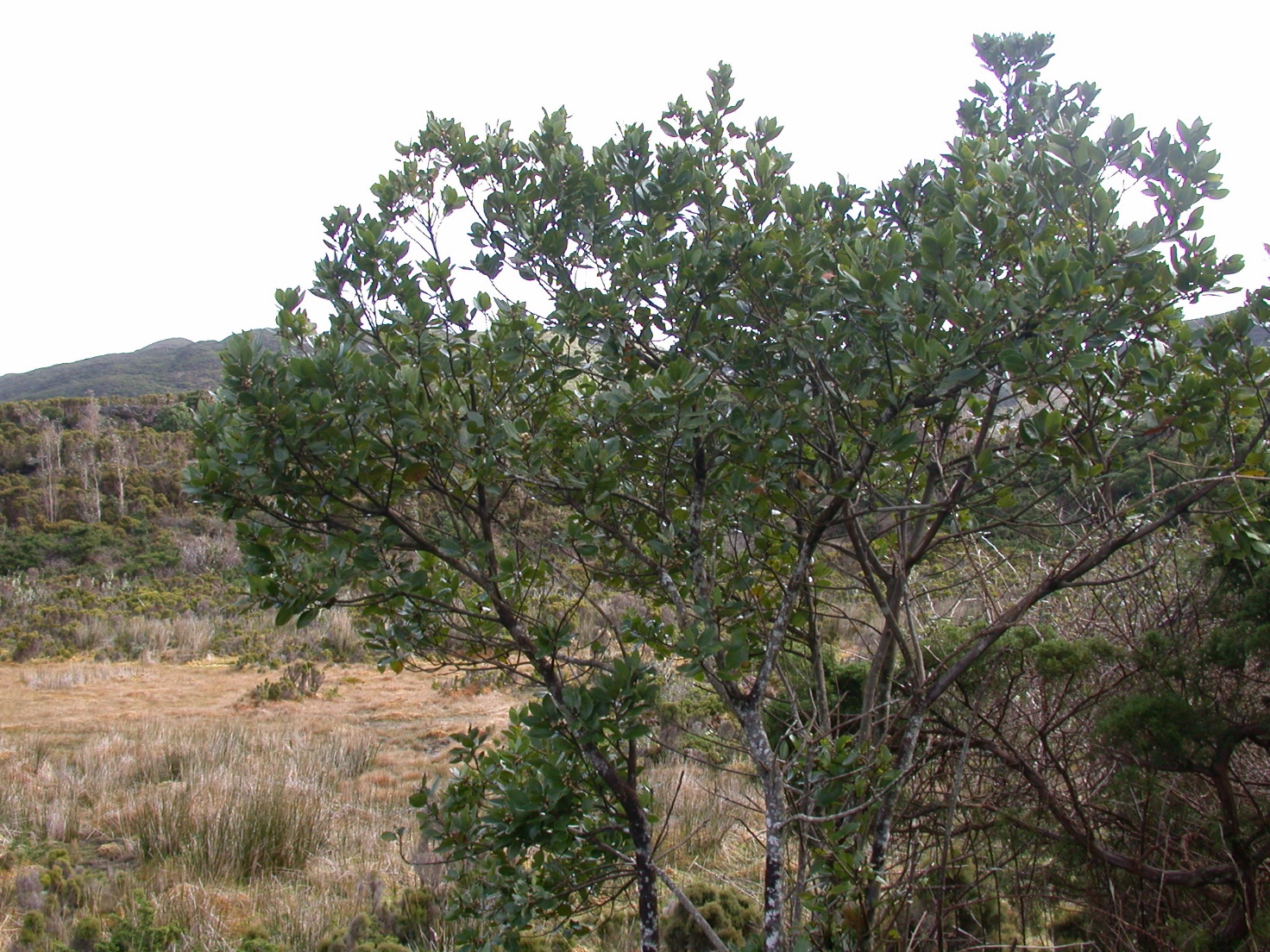


*Vaccinium cylindraceum* – small evergreen tree (up to 4m) with simple, glabrous leaves with up to 5 cm. Occurs in eight Azorean islands (excluding Graciosa) and can be found on native forest patches and on steep slopes.


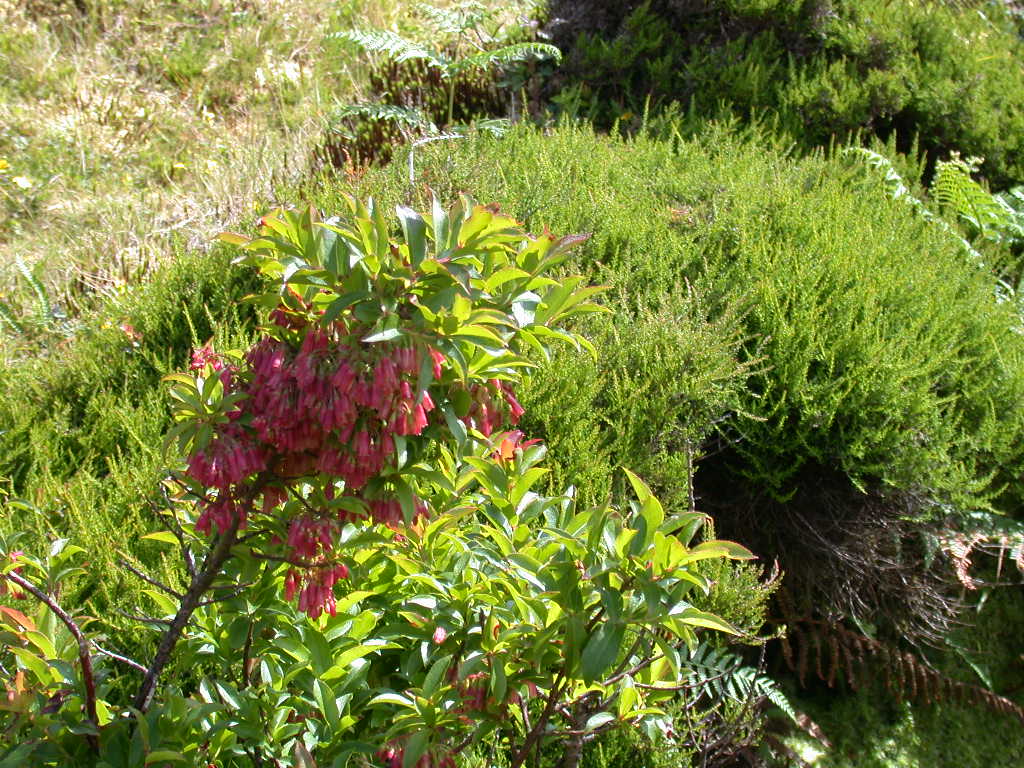

Supplement: S1 Appendix — Brief description and illustration of the study species. (DOCX) [file pone.0219493.s009.docx]
